# T memory stem cell characteristics in autoimmune diseases and their promising therapeutic values

**DOI:** 10.3389/fimmu.2023.1204231

**Published:** 2023-07-11

**Authors:** Pooria Fazeli, Mehdi Kalani, Maryam Hosseini

**Affiliations:** ^1^Trauma Research Center, Shahid Rajaee (Emtiaz) Trauma Hospital, Shiraz University of Medical Sciences, Shiraz, Iran; ^2^Department of Immunology, Prof. Alborzi Clinical Microbiology Research Center, Shiraz University of Medical Sciences, Shiraz, Iran

**Keywords:** T memory stem cell, autoimmune diseases, type 1 diabetes, rheumatoid arthritis, sickle cell disease, hepatitis C virus

## Abstract

Memory T cells are conventionally subdivided into T central memory (T_CM_) and T effector memory (T_EM_) cells. However, a new subset of memory T cells named T memory stem cell (T_SCM_) cells has been recognized that possesses capabilities of both T_CM_ and T_EM_ cells including lymphoid homing and performing effector roles through secretion of cytokines such as interleukin-2 (IL-2) and interferon-gamma (IFN-γ). The T_SCM_ subset has some biological properties including stemness, antigen independency, high proliferative potential, signaling pathway and lipid metabolism. On the other hand, memory T cells are considered one of the principal culprits in the pathogenesis of autoimmune diseases. T_SCM_ cells are responsible for developing long-term defensive immunity against different foreign antigens, alongside tumor-associated antigens, which mainly derive from self-antigens. Hence, antigen-specific T_SCM_ cells can produce antitumor responses that are potentially able to trigger autoimmune activities. Therefore, we reviewed recent evidence on T_SCM_ cell functions in autoimmune disorders including type 1 diabetes, systemic lupus erythematosus, rheumatoid arthritis, acquired aplastic anemia, immune thrombocytopenia, and autoimmune uveitis. We also introduced T_SCM_ cell lineage as an innovative prognostic biomarker and a promising therapeutic target in autoimmune settings.

## 1 Introduction

T cells are identified as key members of the adaptive immune system defending against a wide range of pathogens while making a sharp distinction between self- and non-self-antigens (Ags) (1, 2). The feature “memory” potentiates T cells to produce stronger and more rapid responses during reexposure to the corresponding Ag (1). Memory T cells are also capable of preserving their protectiveness against recognized Ags for several decades without restimulation by those Ags (2, 3). Conventionally, memory T cells are divided into T central memory (T_CM_) and T effector memory (T_EM_) cells according to the expression of their phenotypic markers such as CD45RO/RA, CCR7, and CD62L (3). However, a new subset of memory T cells has been recently recognized during the investigation of the graft-versus-host disease translational model, which possesses features of both T_CM_ and T_EM_ cells including lymphoid homing and effector role performance through secreting effector cytokines like interleukin-2 (IL-2) and interferon-gamma (IFN-γ) named T memory stem cell (T_SCM_) cells. Unlike other memory T-cell subsets, T_SCM_ cells have two main characteristics including stemness and Ag independency along with some biological properties such as high proliferative potential and lipid metabolism (1, 2, 4). Recent investigations extensively reviewed the roles and applications of T_SCM_ cells in malignancies, including melanoma, gastric cancer, B-cell lymphoma, and adult T-cell leukemia as well as infectious disorders such as human immunodeficiency virus type 1 and simian immunodeficiency virus (1–3), but few literatures have focused on properties of T_SCM_ cells in the setting of autoimmune diseases.

An autoimmune disorder is a well-known condition that typically stems from hyperactivation of cells producing inflammatory cytokines accompanied by the disruption of immunoregulatory pathways leading to a persistent response to self-Ags (1). Despite immunosuppressor and anti-inflammatory drugs being routinely used to control autoimmune diseases, no complete success has been achieved with this approach, which can be pertinent to the existence of immune memory cells, specifically T_SCM_ cells (2). Gaining insight into the novel concept of T_SCM_ cells, developing our knowledge regarding T_SCM_ cell’s differentiation, molecular mechanism, signal transduction, and regulation pathways can aid clinicians in designing efficient immunotherapeutic strategies against autoimmune diseases. Therefore, in this study, we review the characteristic features of these innovative memory T cells with their recognized roles in some autoimmune diseases.

## 2 T_SCM_ cells

T_SCM_ cells constitute approximately 2%–3% of circulating T cells (5), with a distinctive gene expression profile that is closely related to that of conventional memory T cells (1, 2, 4). Further investigations showed that T_SCM_ functional roles are apparently different from those of classical memory T subsets (3). T_SCM_ cells emerge mostly in peripheral blood and secondary lymphoid organs (SLOs) and quietly fade at mucosal surfaces (3, 6). Currently, T_SCM_ cells were identified in mice, humans, and nonhuman primates (NHPs) (1–3). According to their life span, T_SCM_ cells are categorized into two subgroups; shorter-lived T_SCM_ cells survive less than 1 year and can be reconstituted rapidly, but another subgroup that is estimated to have at least 9 years of longevity can perfectly preserve their self-renewal and memory abilities even above 25 years (1, 2, 4). The difference between the life span of these two subgroups can be attributed to methylation/demethylation of the promoters of their transcriptional factors, which can switch on/off their self-renewal molecular machinery (1, 4, 7). More importantly, the telomeres of the long-lived T_SCM_ cells are protected by the high levels of telomerase against erosion (1, 2).

### 2.1 T_SCM_ cell differentiation

Different models are proposed on the conversion of T_SCM_ into effector cells. Even though some literature acknowledges the linear model of T_SCM_ cell differentiation (6, 8, 9), circular (on-off-on) and asymmetric models are also suggested (6). Herein, we briefly explain each model and discuss which one is more plausible at least in the context of autoimmune diseases like type 1 diabetes (T1D) (10).

**Figure 1:**
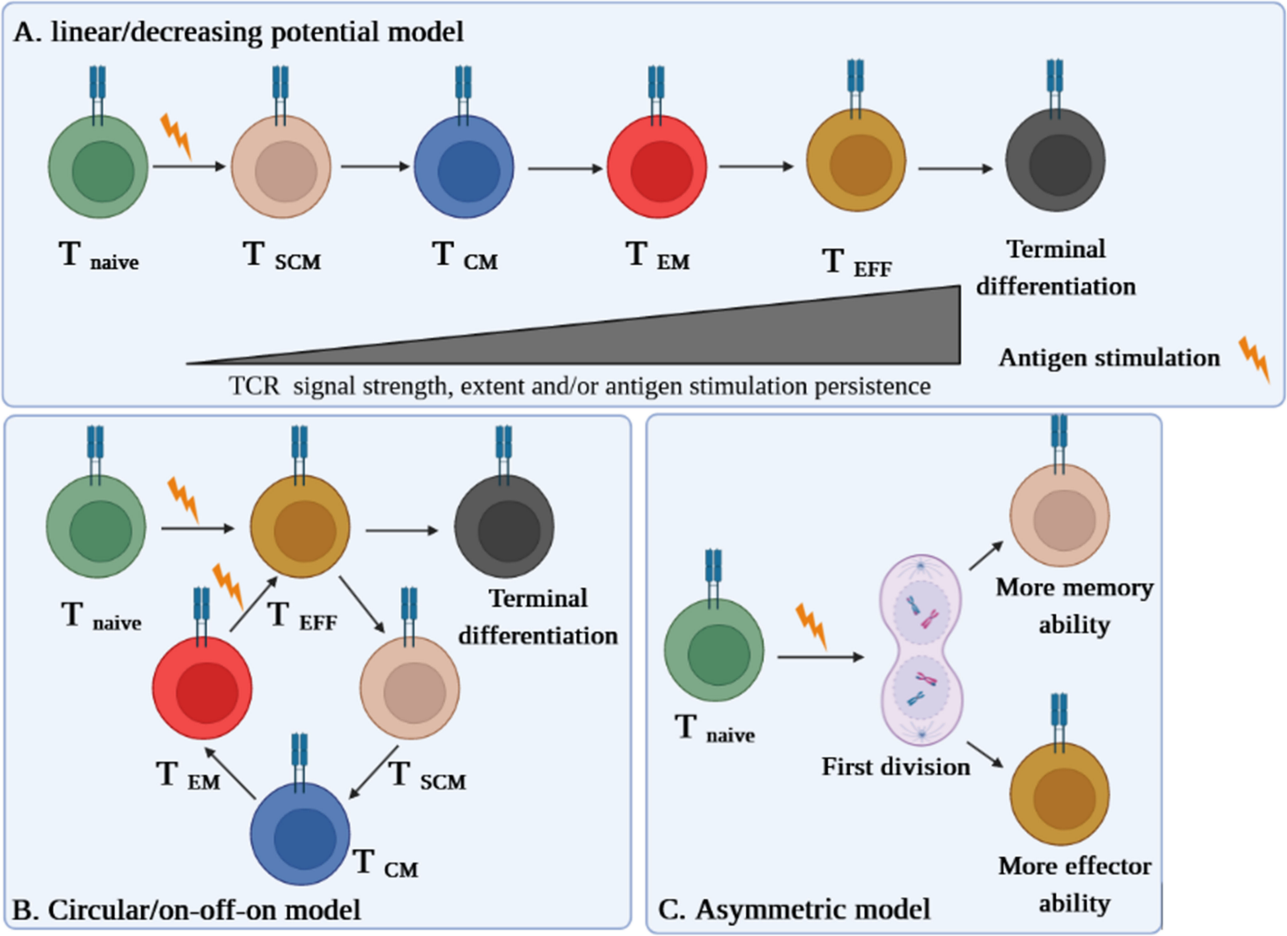
Three suggested models of T_SCM_ cell differentiation. **(A)** The “linear model” stipulates that a T cell loses its memory strength and develops remarkable effector capabilities during differentiation based on the T-cell receptor (TCR) potential signal and extent and persistence of antigenic stimulation on the cells. **(B)** The “circular model” claims that effector T cells should emerge before memory T-cell dedifferentiation. **(C)** The “asymmetric division model” proposes that the formation of memory and effector T cells is predetermined from the first division via asymmetric distribution of key modulators between two daughter cells.

In the linear model, the cell differentiation in each phase depends on the T-cell receptor (TCR) potential signal and extent and persistence of antigenic stimulation on the cells. This model explains that while T cells are directed straightly toward the memory and effector phases, they gradually lose their memory strength and develop remarkably effector capabilities, which is also termed as the “decreasing potential” model (**Figure 1A**). However, it seems that this model is in conflict with the primary definition of T_SCM_ cells in regard to the preservation of memory capacities over long periods of time (6).

The “circular” or “on-off-on” model is opposite the linear one, as it explains that once T cells are exposed to an Ag, they differentiate into effector T cells, and upon the response contraction, the participated effector T cells dedifferentiate into different memory subsets until reencountering with the cognate Ag, by which the cells are able to remember and redifferentiate into the effector T cells (**Figure 1B**). This model could be relatively accepted in the context of some infectious diseases (6).

The third model proposes that the formation of memory and effector T cells is predetermined from the first division via asymmetric distribution of critical transcriptional and epigenetic modulators between two daughter cells whereby one acquires memory ability and another one develops its effector potency. This pattern, called the “asymmetric division model,” can be more conceivable in autoimmune diseases such as T1D in which the full activation signal is received by one daughter cell, while the weak stimulation signal is picked up by another one (**Figure 1C**) (6, 10).

### 2.2 Biological characteristics of T_SCM_ cells

**Figure 2:**
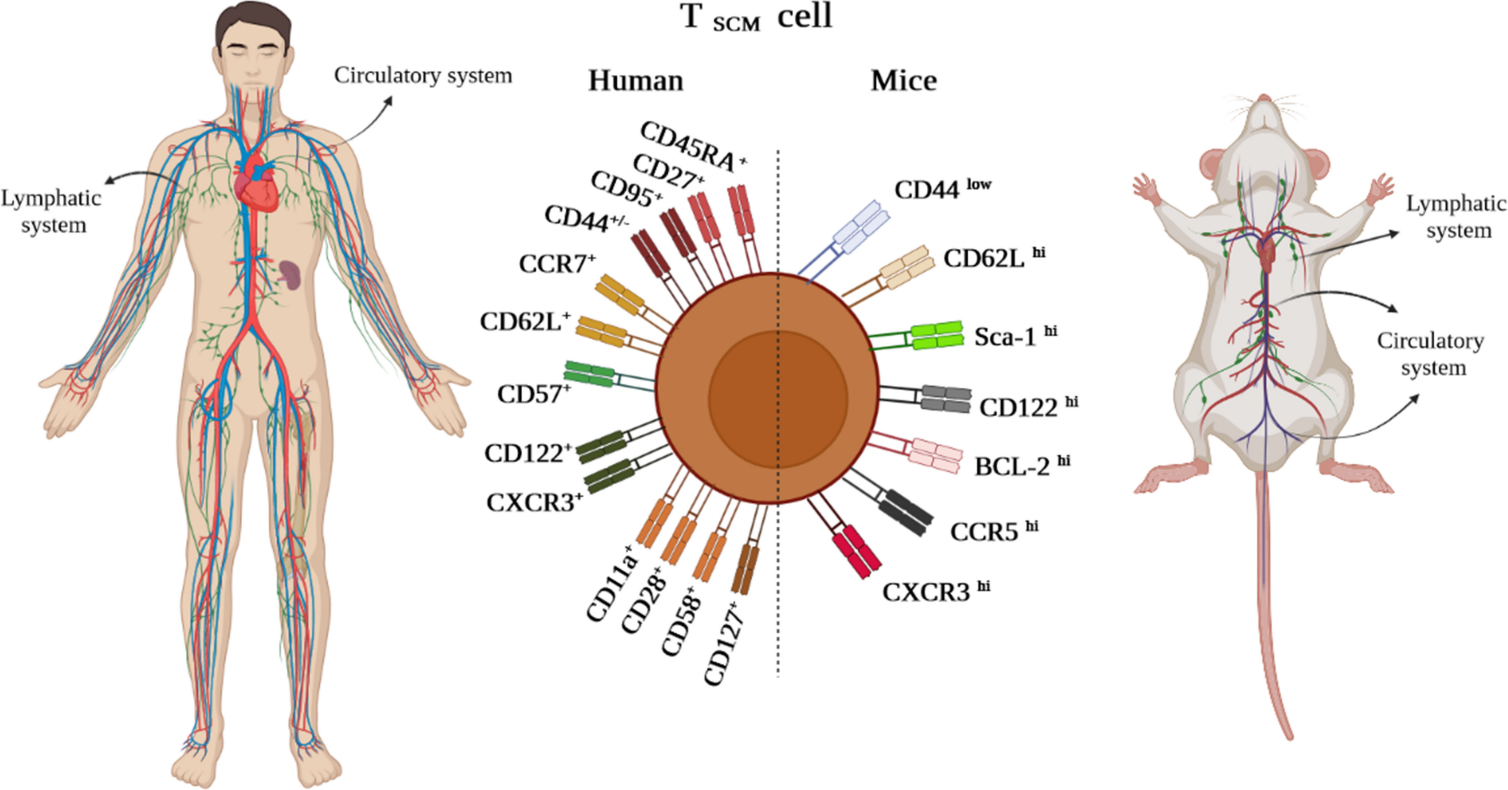
T_SCM_ cell phenotypic expression markers in humans and mice existing in lymphatic and circulatory systems.

Likewise, in other immune cells, immunophenotyping is a helpful technique to identify the T_SCM_ cell subset (3). The subset is characterized in humans and NHPs by expressing the combination of effector and memory T-cell markers, including CD45RA^+^, CD45RO^–^, CD27^+^, CD28^+^, CCR7^+^, CD62L^+^, CD95^+^, CD122^+^, and CD127^+^ (1, 2, 4). Nevertheless, CD45RA^+^, CD45RO^–^, CD27^+^, CD28^+^, CCR7^+^, CD62L^+^, and particularly CD95^+^ are considered distinctive markers between naive and T_SCM_ cells (**Figure 2**) (1, 2, 4). The equivalent markers of T_SCM_ cells in mice are known as CD62L^+^, stem cell marker (Sca-1)^+^, CD122^+^, antiapoptotic marker molecule (Bcl-2)^+^, CCR5^+^, and CXCR3^+^ (**Figure 2**). Given that memory T cells are typically identified by CD45RO^+^ and CD27^+^, contrary to effector T cells expressing CD45RA (1–4), the markers CD27^+^ and CD45RA^+^ by T_SCM_ cells indicate both memory and effector abilities of this subset (4). CCR7 (through binding to CCL19 and CCL21) along with CD62L drive T_SCM_ cells to SLO homing. Not only does CD57 expression reflect telomere shortage (cell senescence) due to repetitive proliferation, but it also enhances either degranulation ability or inflammatory cytokine secretion of T_SCM_ cells (1, 2, 4). Cell imaging techniques showed that normal T cells that express CXCR3 have higher proliferation, multipotency, and polyfunctionality alongside highly released cytokines tumor necrosis factor-alpha (TNF-α), IFN-γ, and IL-2, which are also seen in T_SCM_ cells (2, 4). Obviously, the chemokine receptor helps T_SCM_ cells in lymph node homing (2, 4). Moreover, the expression of CD122 (IL-2R) and CD127 (IL-7R) on T_SCM_ cells indicates that IL-2, IL-15, and IL-7 play critical roles in boosting proliferation and survival of the cells (1–4). The function of T_SCM_ cell human markers has been expressed in **Table 1**.

**Table 1:** The functions of human T_SCM_ cell markers.

CD marker(s)	Function(s)	Ref.
**CD45RA^+^, CD45RO^–^, CD27^+^**	Are related to both memory and effector ability of T_SCM_ cell	(4)
**CCR7^+^, CD62L^+^**	Drive T_SCM_ cell homing in SLO	(1, 2, 4)
**CD57^+^**	1) Telomere shortening (cell senescence) 2) Enhances degranulation ability 3) Enhances inflammatory cytokine secretion	(4)
**CD11α^+^, CD28^+^, CD58^+^**	T cell pan markers	(1–4)
**CD44^+/-^, CD95^+^**	Distinguished markers among naive, effector, and memory T cells	(1–4, 11)
**CD43^-^**	Distinguished marker between effector and memory T cells	(4, 12)
**CD122^+^, CXCR3^+^**	1) Lead to higher proliferation, multipotency, and polyfunctionality 2) Augment the production of TNF-α, IFN-γ, and IL-2 by T_SCM_ cell 3) CXCR3 also helps in T_SCM_ cell LN homing	(4)
**CD127^+^**	Helps in T_SCM_ cell survival and proliferation	(2, 4)

SLO, secondary lymph organ; LN, lymph node; TNF-α, tumor necrosis factor-alpha; IFN-γ, interferon-gamma; IL-2, interleukin-2.

**Figure 3:**
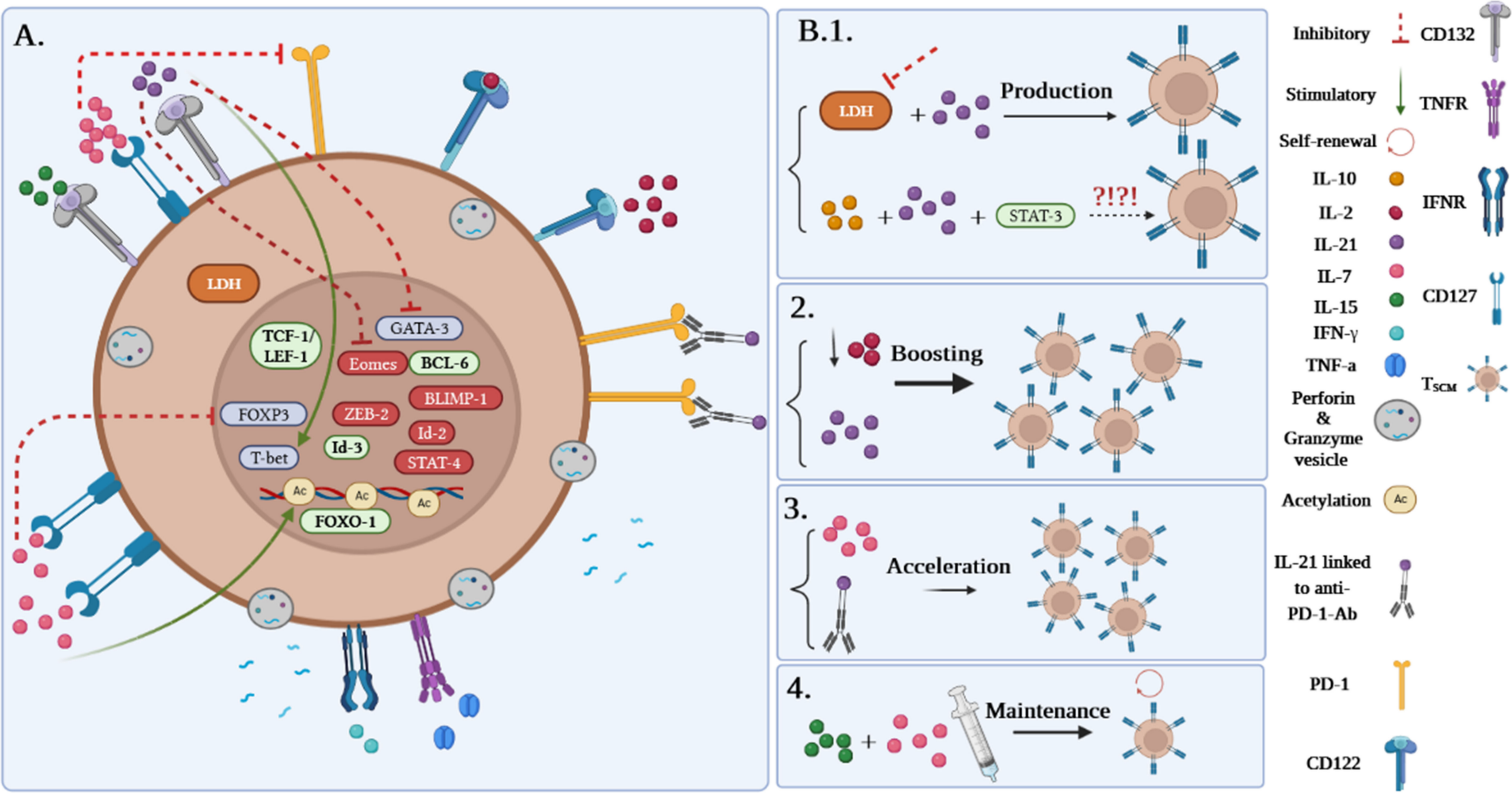
**(A)** Gene profile and cytokine receptor expression on T_SCM_ cells. **(B1–B4)** Different approaches to produce T_SCM_ cells.

Although it has been demonstrated that human T_SCM_ cells are capable of immediately releasing TNF-α, IFN-γ, perforin, and high amounts of IL-2, Zhang et al. demonstrated that murine T_SCM_ cells are not able to produce cytotoxic molecules and IFN-γ (1, 5). Gene expression profiling studies revealed alterations in the genes expressed in human and mouse T_SCM_ cells. Among them, higher expression levels of the transcription factors including TCF-1/LEF, Forkhead box protein O1 (FOXO-1), the inhibitor of DNA binding-3 (Id-3), and B-cell lymphoma-6 (BCL-6) were reported in T_SCM_ cells. In contrast, the levels of T-bet, B lymphocyte-induced maturation protein 1 (BLIMP-1), signal transducer and activator of transcription (STAT)-4, inhibitor of DNA binding-2 (Id-2), Eomes, and zinc finger E-box binding homeobox 2 (ZEB-2) genes were revealed to be partially low (**Figure 3A**). Notably, TCF-1/LEF, Eomes, and Id-3 are regarded as master regulators of the wnt-β-catenin signaling pathway (1, 2, 4, 13).

Among the molecules involved in the development of T_SCM_, it has been suggested that inhibition of lactate dehydrogenase (LDH) along with IL-21 could be effective in this process. Notably, pieces of literature reported that T_SCM_ cells can be generated via the IL-10-IL-21-STAT-3 signaling pathway (**Figure 3B.1**) (4, 14); however, it is unclear how the IL-10-IL-21-STAT-3 signaling pathway plays a proliferative role in this cell subset production. Although IL-2 is defined as the most prevalent growth factor for T cells, it has been demonstrated that high concentrations of IL-2 lead to the expansion of effector T-cell subsets while decreasing early memory T-cell generation through abating T_CM_ cell populations (1, 4). Some studies reported that lower levels of IL-2 in the presence of IL-21 could improve the early memory T-cell proliferation and also could be effective in boosting T_SCM_ cell populations (**Figure 3B.2**) (1, 4). Additionally, IL-7 can accelerate the proliferation of T_SCM_ cells through different mechanisms (**Figures 3A, B.3**). These mechanisms consist of 1) inhibition of programmed cell death-1 (PD-1) and forkhead box p3 (FoxP3) expressions, 2) epigenetic modification through histone acetylation of gene promoters of effector T cells in order to convert to “naive-revertant cells” that can be phenotypically considered T_SCM_ cells, and, more importantly, 3) maintenance of T_SCM_ cell’s phenotype by IL-7 and IL-15 supplementation in the cell culture (**Figure 3B.4**). Similar to IL-7, IL-21 can directly and indirectly enhance T_SCM_ cell populations (4). According to Chen et al., IL-21 coincidently upregulates T-bet and suppressor of cytokine signaling gene expression and downregulates Eomes and GATA binding protein 3 to promote T_SCM_ cell proliferation (**Figure 3A**) (4, 14). Another study has also demonstrated that IL-21 linked to anti-PD-1 antibody (Ab) can prompt T_SCM_ cell development (**Figure 3B.3**) (4, 15).

### 2.3 CD4^+^ and CD8^+^ T _SCM_ cell properties

Human CD8^+^ T_SCM_ cells express not only the surface antigens related to naive T lymphocytes (CD45RO^−^CD62L^+^CCR7^+^) but also markers exclusive for the memory subclass, including CD95, CXCR3, lymphocyte function-associated antigen 1 (LFA-1), and the β chain of IL-2 and IL-15 receptors (16). These cells enjoy similar superior potential for self-renewal and multipotentiality realized in CD4^+^ T_SCM_ (17). CD8^+^ T_SCM_ cells also show a robust response to IL-7 and possess a memory function that causes instant cytokine release after TCR stimulation (18). Meanwhile, from a comparative point of view, *in vivo* studies show that CD4^+^ T_SCM_ cells favorably respond to IL-7, while CD8^+^ T_SCM_ cells are amplified by both IL-7 and IL-15 (19). Gattinoni et al. (20) found that the glycogen synthase kinase-3β (GSK-3β) inhibitor TWS119 or Wnt3a prompts the formation of CD8^+^ T_SCM_ cells through the Wnt/β-catenin/TCF-1 signaling pathway. However, CD8^+^ and CD4^+^ T_SCM_ cells proliferated more efficiently once cocultured with anti-CD3/anti-CD28 conjugated beads alongside low concentrations of IL-7 and IL-15 in comparison with TWS119 exposure (20). Likewise, the generation of CD8^+^ T_SCM_ cells can be improved by IL-21 through the Janus kinase 2 (JAK-2)/STAT-3 pathway (14). Nevertheless, suppressors of cytokine signaling (SOCS) can restrict the impacts of these cytokines. For instance, upon activation of SOCS1, the formation of T_SCM_ cells from naive T cells via IL-21 induction is intensely inhibited (21).

Despite sporadic literature on the characterization of the CD4^+^ subset, a study revealed that CD4^+^ T_SCM_ cells are sensitive to the external environment. That is, during aging and chronic infections, the numbers and the functions of CD4^+^ T_SCM_ cells defect in the circulation. Inflammation can also affect CD4^+^ T_SCM_ cells via the induction of Wnt/β-catenin signaling, which culminates in an improved CD4^+^ T_SCM_ cell proliferative rate. Thus, the T_SCM_ differentiation by promotion of the Wnt/β-catenin pathway with a high concentration of agonist drives the acquisition of a CD4^+^ T_SCM_ phenotype (22).

### 2.4 The metabolism of T_SCM_ cells

Naive T cells are inactive in the peripheral blood and have low metabolic supplies. Thus, they principally use oxidative phosphorylation (OXPHOS) to produce ATP. However, differentiated T cells employ glycolysis to multiply, whereas memory T cells tend to benefit from fatty acid oxidation-dependent OXPHOS to provide ATP, which aids in performing prolonged immune response and heightened longevity (23, 24). Another study on T1D reported that *in vitro* formation of T_SCM_ cells from naive T cells occurred under IL-7 stimulation through overexpression of the glucose transporter GLUT1 to sustain glycolysis and subsequent oxidation of pyruvate in the mitochondria. Thus, targeting glucose metabolism by means of the selective inhibitor for GLUT1 (WZB117) can efficiently diminish the T_SCM_ differentiation in T1D subjects (25). Pilipow et al. (26) displayed that circulating T _SCM_ cells possess an important reservoir of reduced glutathione (GSH) *ex vivo* and limiting ROS with antioxidants in activated CD8^+^ T cells stops terminal differentiation while permitting the generation of long-lived T_SCM_ cells. In this case, N-acetylcysteine was capable of inducing CD8^+^ T_SCM_ cells from naive T precursors *in vitro* (26). In the research conducted by Kondo et al., (27) it has been observed that coculturing T cells with stromal OP9 cells expressing the NOTCH ligand professionally differentiated conventional human T cells into T_SCM_ cells through mitochondrial metabolic reprogramming. NOTCH signaling along with its downstream target, forkhead box M1 (FOXM1), stimulated mitochondrial biogenesis and fatty acid synthesis during T_SCM_ formation, notifying that NOTCH/FOXM1 pathway might be a beneficial target for T_SCM_ cell formation via metabolic alternations (27).

It is stated that imperative transcription factors and cytokines, accompanied by some inhibitors during the process of T-cell differentiation, stimulate T_SCM_ cell production through regulating T cell-related metabolic enzymes (28, 29). Good et al. (30) have declared that blocking the mTOR pathway such as inhibitors of Bruton’s tyrosine kinase (BTK) and IL-2-inducible T-cell kinase (ITK) can direct T cells to T_SCM_ cell differentiation (31). Scholz et al. (31) achieved that inhibition of mTOR complex 1 (mTORC1) by rapamycin or TWS119 in activated human naive T cells eventuates in the induction of T_SCM_ cells via T-cell metabolism alternation toward fatty acid oxidation. As rapamycin is routinely used in the treatment of autoimmune diseases (32), it seems that the usage of rapamycin may adversely exaggerate autoimmune diseases in the long run. Additionally, recent evidence indicates that T_SCM_ cells can be induced by Mek1/2 inhibitor (Meki) through regulating the metabolism regardless of affecting TCR-mediated activation (33). These studies point out that the regulation of metabolism and glycolysis is the fundamental factor in prompting the T_SCM_ formation. Hence, targeted metabolic checkpoints can bring about T cells differentiating into memory and afford more fresh T cells for immunotherapy.

## 3 T_SCM_ cell in autoimmune diseases

Undoubtedly, memory T cells are one of the principal culprits in the pathogenesis of autoimmune diseases. T_SCM_ cells are responsible for developing long-term defensive immunity against different foreign Ags involving viral, bacterial, parasitic, and, in particular, tumor-associated Ags (1, 4). Since tumor-associated Ags mainly derive from self-Ags, once T_SCM_ cells trigger antitumor responses, it can ultimately cause autoimmune diseases (1, 2, 4). Notably, Hosokawa et al. (34) and our team separately showed that T_SCM_ cells are the least exhausted population than other memory T-cell subsets, which may be due to self-renewal potency and possessing high-length telomeres (34, 35). This evidence may justify the chronic and progressive hallmarks of autoimmune disorders. Meanwhile, Hosokawa et al. (34) exposed that upregulation of PD‐1 on CD8^+^ T_SCM_ cells in aplastic anemia patients parallel to elevated IFN-γ secretion could be an indicator of autoreactive CD8^+^ T_SCM_ cell’s clonal expansion. PD‐1, as one of the core costimulatory molecules (36), is expressed on various immune cells, in particular, exhausted and activated T cells to derive inhibitory signals, modulate T-cell response, and maintain peripheral tolerance (36, 37). Recent studies demonstrated that CD4^+^ and/or CD8^+^ T_SCM_ cells play a vital role in the pathogenesis of autoimmune diseases such as systemic lupus erythematosus (SLE) (34, 38), aplastic anemia (AA) (34), autoimmune uveitis (34), T1D (25, 35), rheumatoid arthritis (RA) (39, 40), and immune thrombocytopenia (ITP) (41). However, the question whether the increased frequency of T_SCM_ cells results from immune activation or *vice versa* remains unanswered. Most investigators in the context of autoimmune diseases consistently speculated that due to the ability of T_SCM_ cells to recreate all memory and effector T-cell subsets, the increased frequency of T_SCM_ cells could lead to autoimmune disease progression. Therefore, T_SCM_ cell subsets can be a potential biomarker for autoimmune diseases and their response prediction (25, 34, 35, 38–41). Here, we explain the role of T_SCM_ cells in some autoimmune diseases (**Table 2**).

**Table 2:** The role of CD4^+^ and/or CD8^+^ T_SCM_ in the pathogenesis of autoimmune diseases.

First author (Year)	Patient	HC	Method	Results	Conclusion	Ref.
	(N)					
**Hosokawa (2016)**	55 AA	41	Flowcytometry	CD8^+^ T_SCM_ cells in AA > HC	The role of T_SCM_ cells in the regulation of AID pathogenesis	(34)
	34 AU			CD8^+^ T_SCM_ cells in uveitis > HC		
	43 SLE			CD4^+^ T_SCM_ cells in SLE > HC		
	5 SCD			CD8^+^ T_SCM_ cells in SCD > HC		
**Lee (2018)**	65 SLE	72	Flowcytometry qRT-PCR ELISA	CD4^+^ and CD8^+^ T_SCM_ cells in SLE > HC	The role of T_SCM_ cells in the pathogenesis of SLE by maintaining TFH cells	(38)
**Vignali (2018)**	14 T1D	16	Flowcytometry CSFE proliferation assay Confocal microscopy	Ag-specific CD8^+^ T_SCM_ cells in T1D > HC	Long-lived autoreactive T_SCM_ cells can be considered as a reservoir of pathogenic effector T cells exacerbating T1D	(25)
**Fazeli (2022)**	30 T1D	15	Flowcytometry	CD4^+^ T_SCM_ cells in T1D > HC	Regarding the capacities of T_SCM_ cells to create all memory and effector subsets, their high frequency aggravates the disease.	(35)
**Takeshita (2019)**	311 RA	73	Flowcytometry RNA sequencing	CD4^+^ and CD8^+^ T_SCM_ cells in RA > HC	T_SCM_ cells contribute to the RA pathogenesis by producing pathogenic T cells with self-renewal	(40)
**Cianciotti (2020)**	27 RA	20	Flowcytometry HLA-typing	Cit-vimentin–specific CD4^+^ T_SCM_ cells in RA > HC	Increased Cit-vimentin–specific CD4^+^ T_SCM_ cells in RA patients is not exposed to TNF-α blockade and might be involved in the natural history of the disease	(39)
**Cao (2019)**	20 ITP	26	Flowcytometry	CD8^+^ T_SCM_ cells in ITP > HC	CD8+ T_SCM_ cells cause the disease progression	(41)

HC, healthy control; AA, acquired aplastic anemia; AU, autoimmune uveitis; SLE, systemic lupus erythematosus; SCD, sickle cell disease; T1D, type 1 diabetes; RA, rheumatoid arthritis; ITP, immune thrombocytopenia; qRT-PCR, quantitative real-time PCR; HLA, human leukocyte antigen; AID, autoimmune disease; TFH, T follicular helper cell; T_SCM,_ T memory stem cell.

### 3.1 T_SCM_ cells and AA

Acquired AA is a rare condition of bone marrow failure syndrome in which hematopoietic stem/progenitor cells (HSPCs) are destroyed by mechanisms unrelated to the inherited syndrome (42). Despite the exact pathophysiology mechanism of AA still being blurred and the specificity of some recognized auto-Ags such as diazepam-binding related protein-1 not being proven *in vivo*, the immune attack to allogeneic hematopoietic cells by autoreactive T cells is considered as an underlying mechanism of autoimmunity in AA (43). Some features of autoreactive T cells in AA encouraged Hosokawa et al. (34) to explore the role of T_SCM_ cells in the immunopathogenesis of AA. It has been shown that the recognition of HSPC-restricted Ags through major histocompatibility complex (MHC) class I or II by oligoclonal CD8^+^ autoreactive T cells leads to pro-inflammatory cytokine secretion like IFN-γ against HSPC cells. Following immunosuppressive therapy (IST) with anti-thymocyte globulin (ATG) and cyclosporine A (CsA), the regeneration of oligoclonal T cells and even the new ones can occur (34). Accordantly, Hosokawa et al. (34) displayed that, in comparison to healthy individuals, the rate of CD8^+^ T_SCM_ cells in AA patients was higher at the onset of diagnosis and after IST in responder (complete and partial response) and non-responder patients, respectively. This indicates a favorable response to IST when CD8^+^ T_SCM_ cell frequency is high at the time of diagnosis in responders and inversely bringing about disease aggravation in non-responders to IST. Moreover, their intracellular staining revealed that both CD4^+^ and CD8^+^ T_SCM_ cells have more elevated levels of IL-2 and IFN-γ than those in healthy controls (34).

### 3.2 T_SCM_ cells and autoimmune uveitis

AU is an organ-specific disorder in which immune cells and, in particular, CD4^+^ and CD8^+^ Ag-specific memory T cells reside within the ocular tissue (44). The study of Hosokawa et al. (34) on AU patients showed that CD8^+^ T_SCM_ cell frequencies in these patients are significantly higher than those in healthy individuals. They also displayed that IST (combination of prednisolone and anti-TNF-α antibody) can be effective in reducing the CD8^+^ T_SCM_ cell population in patients with AU (34), suggesting CD8^+^ T_SCM_ cells as a potential marker associating with a better response to IST following lower frequency of the cells.

### 3.3 T_SCM_ cells and SLE

Systemic lupus erythematosus (SLE) is a complex autoimmune disease affecting multiple organs. Despite that the pathogenesis of SLE is still not fully understood, it is believed that factors, including environmental, hormonal, genetic, and immunological, serve a role in SLE development. Among others, the role of immunological dysregulation seems more prominent. In fact, immunological dysregulation can disrupt the balance of T helper (TH)1/TH2 and TH17/T regulatory (Treg) cell and ultimately shift them toward autoreactive T cells (45). T follicular helper (TFH) cells also interact with B cells, to boost their autoantibody (auto-Ab) production (38, 45). Auto-Abs and autoreactive T-cell activity are considered the major hindrance to achieving complete remission in SLE patients, despite long-lasting IST that indicates the emergence of the T_SCM_ cell population in SLE patients (38, 45). Recently, Hosokawa et al. (34) demonstrated that the CD4^+^ T_SCM_ cell population in SLE patients is lower than that in healthy controls due to receiving IST during sampling (34). Furthermore, Lee et al. (38) found that CD4^+^ and CD8^+^ T_SCM_ cell frequencies in SLE patients were remarkably elevated than those in the controls. They also observed that the CD4^+^ T_SCM_ cells of SLE patients can differentiate into TFH cells through BCL6, CXCR5, PD1, ICOS, LEF1, TCF-1, and IL-21 (as TFH cell inducer) gene overexpression, and BLIMP-1 gene encoding downregulation, leading to pathogenic auto-Ab formation (38). Strikingly, they declared that the CD4^+^ T_SCM_ cell population in SLE patients participates in the inflammatory process by producing higher levels of TNF-α, IFN-γ, IL-2, and IFN-α than those in normal individuals (38).

### 3.4 T_SCM_ cells and ITP

ITP is an acquired autoimmune disease that is characterized by platelet devastation due to T-cell dysfunction (46). Indeed, autoreactive T cells, including TH1 and TH17, give rise to auto-Ab production against platelets, and cytotoxic CD8^+^ T cells invade the surface glycoprotein GPIIb/IIIa of platelets. Additionally, the number and function of CD4^+^CD25^+^Treg cells decrease, resulting in the development and progression of ITP (46). In the investigation conducted by Cao et al., (41) it was disclosed that the population of CD8^+^ T_SCM_ cells in ITP patients outnumbered that of controls. Moreover, they found that prednisolone prescription, an IST, reduces the CD8^+^ T_SCM_ cell frequency and alleviates platelet destruction in responder groups (complete and partial) (41). They suggested that the higher frequency of this T-cell memory subset can lead to ITP exacerbation (41).

### 3.5 T_SCM_ cells and T1D

**Figure 4:**
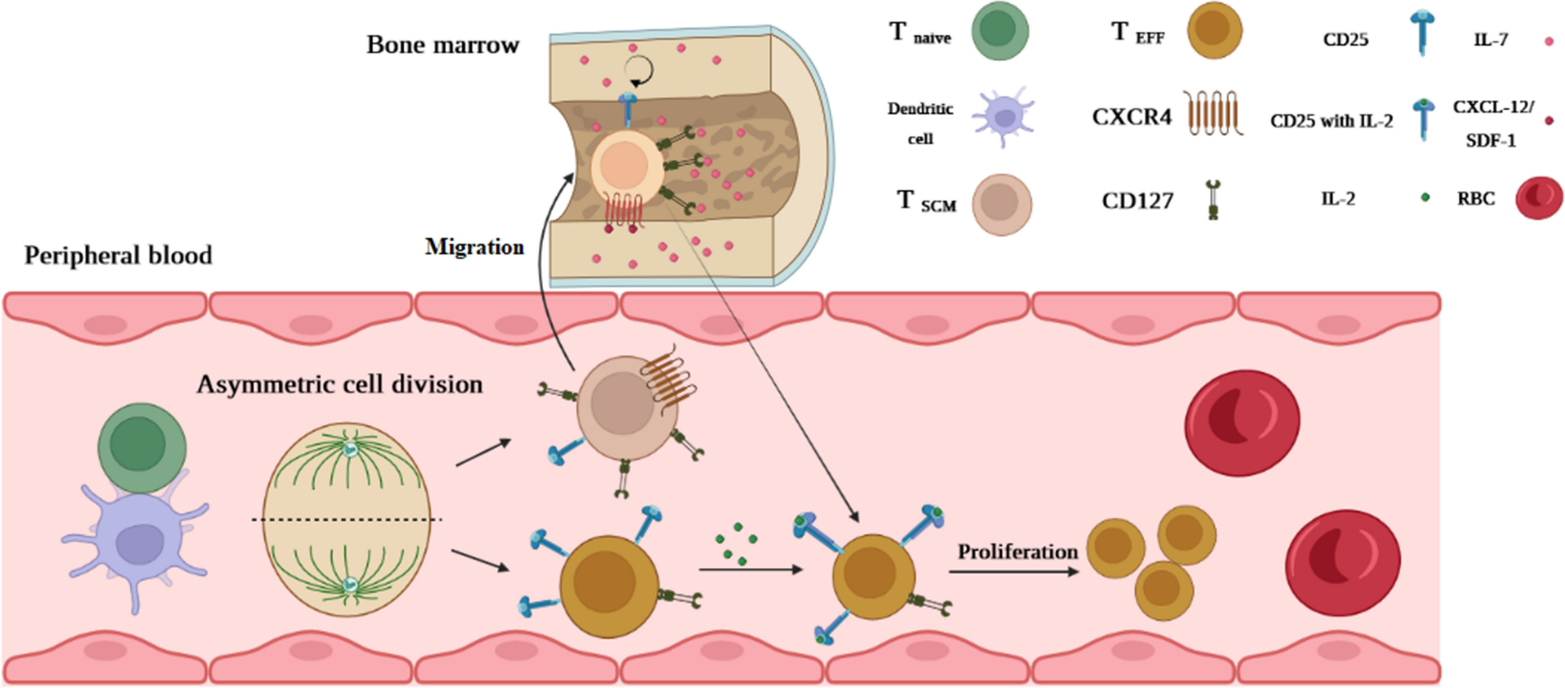
The possible mechanism of T_SCM_ cell generation in T1D. An activated T cell undergoes mitosis coincidentally in the immune synapse forming between DC and naive T cells. After polarization, one of which becomes a fully activated daughter cell (because of permanent interaction with DC and receiving stimulatory signals) differentiating into CD25^hi^ CD127^low^ effector T cells. Another daughter cell (resting cell) without receiving a full-activation signal expressing CD25^low^ CD127^hi^ (T_SCM_ cell) migrates via chemokine receptor CXCR-4 upregulation linking to CXCL-12 into the bone marrow (BM). IL-7 in BM assists T_SCM_ cells in self-renewal by reducing their turnover. Once a second Ag stimulation occurs, T1D T_SCM_ cells that arrested in BM can reconstitute specific effector and memory autoreactive T cells.

T1D is another chronic autoimmune disease in which autoreactive T cells attack β-cell auto-Ags such as glutamic acid decarboxylase 65 (GAD65), (pro)insulin, and islet-specific glucose-6-phosphatase catalytic subunit-related protein (IGRP) (25). Some autoreactive T cells in T1D patients exhibit distinguishable characteristics, including memory marker expression like CD95 and the presence of IL-7, which prolongs the survival and maintenance of autoreactive T cells, particularly T_SCM_ cells. More importantly, similar to other autoimmune diseases, a high dosage of IST during and after pancreas transplantation fails to eliminate autoreactive memory T cells in these patients (25). The mechanism of T_SCM_ cells in T1D is explainable by the “asymmetric division model” by which activation of T cells through β-cell auto-Ag presentation in an immune synapse between DC and naive T-cell undertakes mitosis simultaneously in the immune synapse. After polarization, the daughter cell receiving a continuous strong signal from the immune synapse becomes fully activated and consequently differentiates into CD25^hi^ CD127^low^ effector T cell (6). Another daughter cell with a faint signal expressing CD25^low^ CD127^hi^ (T_SCM_ cell) migrates via chemokine receptor CXCR-4 upregulation linking to CXCL-12 (stromal derived factor-1) into bone marrow (BM) where the stromal cells immensely produce IL-7 (6). A hemostatic cytokine IL-7 not only upregulates CXCR-4 on T cells but also assists T_SCM_ cells in self-renewal. It is postulated that the T1D autoreactive T cells arrested in BM and decreased their turnover by IL-7 can reconstitute specific effector and memory autoreactive T cells (**Figure 4**) (6). Vignali et al. (25) measured the frequency of autoreactive CD8^+^ T_SCM_ cells against GAD65, insulin, and IGRP in new-onset (<6 months T1D) and long-term (>20 years T1D) patients. They observed that the frequency of circulating specific CD8^+^ T_SCM_ cells against GAD65 and insulin in the new-onset patients was significantly higher than that in healthy controls (25). They also found that IL-7 can considerably amplify their frequency by GLUT-1 upregulation in T1D patients compared to the controls (25). Our study revealed that in new-onset (<1 year T1D), the frequency of CD4^+^ T_SCM_ cells is noticeably higher than that in long-term (>5 years T1D) and normal individuals (35). Therefore, both studies stated that T_SCM_ cell subsets can lead to disease progression (25, 35).

### 3.6 T_SCM_ cells and RA

RA is a chronic inflammatory autoimmune disease in which synovial fluid (SF) is predominantly targeted by autoreactive T cells and leads to arthropathy (47). Besides autoreactive CD4^+^ T cells serving a key role in the immunopathogenesis of RA through their TH1, TH17, and TFH cell subsets, autoreactive CD8^+^ T cells are observed in the SF of new-onset patients (40). The study performed by Takeshita et al. (40) showed that the frequency of CD4^+^ and CD8^+^ T_SCM_ cells elevated in RA patients’ peripheral blood and SF. Moreover, they found that among immunosuppressant drugs, not only does methotrexate (MTX) decline T_SCM_ cell frequency, but it also reduces other T-cell subsets (except for TFH cells), resulting in MTX’s suppression effect on T-cell proliferation pathways including E2F, IL-2-STAT5, and mTORC1 (40). Consistent with this study, Cianciotti et al. (39) displayed that the citrullinated vimentin (Cit-vimentin)-specific CD4^+^ T_SCM_ cell population is higher in the circulation of RA patients than that in controls. They also found that TNF-α blockade can attenuate this subset frequency and prohibit differentiation of circulating TH17 from CD4^+^ T_SCM_ cells through blocking TNFII receptor (TNFRII) signaling, suggesting TNF-α as a prosurvival factor for T_SCM_ cells. Thereby, both mentioned studies implicated that the frequency of T_SCM_ cells can be a beneficial marker for disease development and response prediction (39).

## 4 T_SCM_ cells and hepatitis C virus

Chronic hepatitis C virus (HCV) is one of the most important viruses related to autoimmune diseases that can inflict destructive effects on the liver, thyroid tissue, and platelets (48). Although the disease etiology is still not clarified, T_SCM_ cells, as a less-differentiated memory T-cell subset, play a fundamental role in the long-term immune defense against HCV (49, 50). Lu et al. (49) observed that the CD8^+^ T_SCM_ cell population was raised in both monoinfected HCV and coinfected HCV/HIV patients. Their investigation also showed that the CD8^+^ T_SCM_ cell population respectively has direct and indirect correlations with T_CM_ cells and T_EM_ cells, which can help maintain T-cell hemostasis (49). Moreover, they reported that a high incidence of CD8^+^ T_SCM_ cells can effectively control HCV replication in monoinfected HCV patients, indicating that the CD8^+^ T_SCM_ cell population has a protective impact in HCV infection and paving the way for T_SCM_ cell-based vaccine design to attain HCV clearance (49).

## 5 T_SCM_ cells and sickle cell disease

Sickle cell disease (SCD) is a nonspecific chronic inflammation that may occur due to environmental factors like transfusions leading to red blood cell (RBC) deformation, hemolysis, and vaso-occlusion development (34, 51, 52). Indeed, the physicochemical alterations of RBCs culminate in hemolysis and erythrocyte rupture, which in turn trigger the inflammatory responses and lymphocyte activation through necrotic particle production (52). Although SCD is not recognized as an autoimmune disease, Hosokawa et al. (34) surprisingly observed that the CD8^+^ T_SCM_ cell frequency in SCD was significantly higher in comparison with controls despite their limited sample size (five patients). This can be justified by previous studies that demonstrated that permanent inflammation in SCD likely induces memory T-cell formation alongside various pro-inflammatory and inflammatory cytokines such as IL-2, IL-7, and IL-15 (51), requiring cytokines for CD8^+^ T_SCM_ cell generation (1, 4). Meanwhile, future studies are essential to definitively prove our explanation.

## 6 Therapeutic outlooks

The emerging role of T_SCM_ cells in the pathogenesis of autoimmune diseases presents new opportunities for prevention or even treatment of these diseases. Eliminating T_SCM_ cells, which are detected in high levels in various autoimmune disorders, can improve the efficacy of immunosuppressive therapeutics and alleviate autoreactive symptoms. Molecular regulation of the proliferation, metabolic behavior, and self-renewal of T_SCM_ cells can provide promising targets for treating autoimmune illnesses. In this regard, pharmaceutical inhibition of Wnt-β-catenin signaling, which is a crucial driver for the induction of T_SCM_ cells (20), might limit the expansion of these cells. Notwithstanding, few attempts have been made to target these key molecules in research. Implicating current treatment approaches, including adoptive cell transfer and gene therapy, will be noteworthy in the setting of targeted therapy of molecules restricting T_SCM_ cell generation. Ongoing studies might open new doors in this era for the treatment of autoimmune diseases.

On the other side, it is unclear what the limitations of manipulating T_SCM_ cells are in the setting of autoimmune diseases. Some questions that will arise include whether downregulation of these cells to treat autoimmunity induces detrimental effects such as the development of tumors and infections or how much these cells should be reduced and after how long will the amount of memory cells be restored in the context of various autoimmune diseases. Whether the manipulation of T_SCM_ cells will affect the function of other T cells in the circulation. It is speculated that targeting of T_SCM_ cells in autoimmune diseases is a form of personalized medicine, which can be prescribed based on the patient’s age, weight, and disease condition. Further experiments are necessary to answer various questions surrounding the targeting of T_SCM_ cells in autoimmune diseases.

## 7 Conclusion

T_SCM_ cells possess a unique capability for enhanced self-renewal and multidifferentiation along with performing effector functions. Given that T_SCM_ cells are newly discovered T cells that play roles in autoimmune diseases, gaining a deep understanding of their importance in the development and progression of autoimmune disorders may be at the forefront of research interests. Future attempts are also needed to analyze transcriptome profiles and effector molecules of T_SCM_ cells, with a precise exploration of pathways determining T-cell differentiation and function, and suggest strategies targeting specific molecules in the control or treatment of autoimmune disorders.

## Author contributions

All authors contributed to the article and approved the submitted version.

## References

[1] Gao S Liang X Wang H Bao B Zhang K Zhu Y. Stem cell-like memory T cells: a perspective from the dark side. Cell Immunol (2021) 361:104273. doi: 10.1016/j.cellimm.2020.104273 33422699

[2] Wang Y Qiu F Xu Y Hou X Zhang Z Huang L. Stem cell-like memory T cells: the generation and application. J Leukoc Biol (2021) 110(6):1209–23. doi: 10.1002/JLB.5MR0321-145R 34402104

[3] Gattinoni L Speiser DE Lichterfeld M Bonini C. T Memory stem cells in health and disease. Nat Med (2017) 23(1):18–27. doi: 10.1038/nm.4241 28060797 PMC6354775

[4] Li Y Wu Y Yang X Zhou S. Immunotherapeutic potential of T memory stem cells. Front Oncol (2021) 11:723888. doi: 10.3389/fonc.2021.723888 34604060 PMC8485052

[5] Zhang Y Joe G Hexner E Zhu J Emerson SG. Host-reactive CD8+ memory stem cells in graft-versus-host disease. Nat Med (2005) 11(12):1299–305. doi: 10.1038/nm1326 16288282

[6] Kedzierska K Koutsakos M. The ABC of major histocompatibility complexes and T cell receptors in health and disease. Viral Immunol (2020) 33(3):160–78. doi: 10.1089/vim.2019.0184 PMC7185345 32286182

[7] Abdelsamed HA Moustaki A Fan Y Dogra P Ghoneim HE Zebley CC. Human memory CD8 T cell effector potential is epigenetically preserved during in vivo homeostasis. J Exp Med (2017) 214(6):1593–606. doi: 10.1084/jem.20161760 PMC5461005 28490440

[8] Buchholz VR Schumacher TN Busch DH. T Cell fate at the single-cell level. Annu Rev Immunol (2016) 34:65–92. doi: 10.1146/annurev-immunol-032414-112014 26666651

[9] Henning AN Roychoudhuri R Restifo NP. Epigenetic control of CD8+ T cell differentiation. Nat Rev Immunol (2018) 18(5):340–56. doi: 10.1038/nri.2017.146 PMC6327307 29379213

[10] Monti P Heninger A-K Bonifacio E. Differentiation, expansion, and homeostasis of autoreactive T cells in type 1 diabetes mellitus. Curr Diab Rep (2009) 9(2):113–8. doi: 10.1007/s11892-009-0020-y 19323955

[11] Schumann J Stanko K Schliesser U Appelt C Sawitzki B. Differences in CD44 surface expression levels and function discriminates IL-17 and IFN-γ producing helper T cells. PLoS One. (2015) 10(7). doi: 10.1371/journal.pone.0132479 PMC4501817 26172046

[12] Lee J-B Chang J. CD43 expression regulated by IL-12 signaling is associated with survival of CD8 T cells. Immune Netw (2010) 10(5):153–63. doi: 10.4110/in.2010.10.5.153 PMC2993947 21165244

[13] Zehn D Thimme R Lugli E de Almeida GP Oxenius A. ‘Stem-like’precursors are the fount to sustain persistent CD8+ T cell responses. Nat Immunol (2022) 23(6):836–47. doi: 10.1038/s41590-022-01219-w 35624209

[14] Chen Y Yu F Jiang Y Chen J Wu K Chen X. Adoptive transfer of interleukin-21-stimulated human CD8+ T memory stem cells efficiently inhibits tumor growth. J Immunother (Hagerstown Md: 1997) (2018) 41(6):274. doi: 10.1097/CJI.0000000000000229 PMC6012057 29864078

[15] Li Y Cong Y Jia M He Q Zhong H Zhao Y. Targeting IL-21 to tumor-reactive T cells enhances memory T cell responses and anti-PD-1 antibody therapy. Nat Commun (2021) 12(1):951. doi: 10.1038/s41467-021-21241-0 33574265 PMC7878483

[16] Gattinoni L Lugli E Ji Y Pos Z Paulos CM Quigley MF. A human memory T cell subset with stem cell–like properties. Nat Med (2011) 17(10):1290–7. doi: 10.1038/nm.2446 PMC3192229 21926977

[17] Takeshita M Suzuki K Kassai Y Takiguchi M Nakayama Y Otomo Y. Polarization diversity of human CD4+ stem cell memory T cells. Clin Immunol (2015) 159(1):107–17. doi: 10.1016/j.clim.2015.04.010 25931384

[18] Bishop EL Gudgeon N Dimeloe S. Control of T cell metabolism by cytokines and hormones. Front Immunol (2021) 12:653605. doi: 10.3389/fimmu.2021.653605 33927722 PMC8076900

[19] Lugli E Gattinoni L Roberto A Mavilio D Price DA Restifo NP. Identification, isolation and *in vitro* expansion of human and nonhuman primate T stem cell memory cells. Nat Protoc (2013) 8(1):33–42. doi: 10.1038/nprot.2012.143 23222456 PMC6328292

[20] Gattinoni L Zhong X-S Palmer DC Ji Y Hinrichs CS Yu Z. Wnt signaling arrests effector T cell differentiation and generates CD8+ memory stem cells. Nat Med (2009) 15(7):808–13. doi: 10.1038/nm.1982 PMC2707501 19525962

[21] Palmer DC Restifo NP. Suppressors of cytokine signaling (SOCS) in T cell differentiation, maturation, and function. Trends Immunol (2009) 30(12):592–602. doi: 10.1016/j.it.2009.09.009 19879803 PMC2787651

[22] Kared H Tan SW Lau MC Chevrier M Tan C How W. Immunological history governs human stem cell memory CD4 heterogeneity via the wnt signaling pathway. Nat Commun (2020) 11(1):821. doi: 10.1038/s41467-020-14442-6 32041953 PMC7010798

[23] Pearce EL Walsh MC Cejas PJ Harms GM Shen H Wang LS. Enhancing CD8 T-cell memory by modulating fatty acid metabolism. Nature (2009) 460(7251):103–7. doi: 10.1038/nature08097 PMC2803086 19494812

[24] Lécuyer E Rakotobe S Lengliné-Garnier H Lebreton C Picard M Juste C. Segmented filamentous bacterium uses secondary and tertiary lymphoid tissues to induce gut IgA and specific T helper 17 cell responses. Immunity (2014) 40(4):608–20. doi: 10.1016/j.immuni.2014.03.009 24745335

[25] Vignali D Cantarelli E Bordignon C Canu A Citro A Annoni A. Detection and characterization of CD8+ autoreactive memory stem T cells in patients with type 1 diabetes. Diabetes (2018) 67(5):936–45. doi: 10.2337/db17-1390 29506985

[26] Pilipow K Scamardella E Puccio S Gautam S Paoli FD Mazza EM. Antioxidant metabolism regulates CD8+ T memory stem cell formation and antitumor immunity. JCI Insight (2018) 3(18):e122299. doi: 10.1172/jci.insight.122299 30232291 PMC6237218

[27] Kondo T Ando M Nagai N Tomisato W Srirat T Liu B. The NOTCH–FOXM1 axis plays a key role in mitochondrial biogenesis in the induction of human stem cell memory–like CAR-T CellsNotch–FOXM1 axis induces stem cell memory–like CAR-T cells. Cancer Res (2020) 80(3):471–83. doi: 10.1158/0008-5472.CAN-19-1196 31767627

[28] Hu Z Zou Q Su B. Regulation of T cell immunity by cellular metabolism. Front Med (2018) 12:463–72. doi: 10.1007/s11684-018-0668-2 30112717

[29] Mousset CM Hobo W Ji Y Fredrix H De Giorgi V Allison RD. Ex Vivo AKT-Inhibition Facilitates Generation of Polyfunctional Stem Cell Memory-Like CD8(+) T Cells for Adoptive Immunotherapy. Oncoimmunology (2018) 7(10):e1488565. doi: 10.1080/2162402X.2018.1488565 30288356 PMC6169586

[30] Good Z Borges L Vivanco Gonzalez N Sahaf B Samusik N Tibshirani R. Proliferation tracing with single-cell mass cytometry optimizes generation of stem cell memory-like T cells. Nat Biotechnol (2019) 37, 259–266. doi: 10.1038/s41587-019-0033-2 30742126 PMC6521980

[31] Scholz G Jandus C Zhang L Grandclément C Lopez-Mejia IC Soneson C. Modulation of mTOR signalling triggers the formation of stem cell-like memory T cells. EBioMedicine (2016) 4:50–61. doi: 10.1016/j.ebiom.2016.01.019 26981571 PMC4776068

[32] Xiang M Kim H Ho VT Walker SR Bar-Natan M Anahtar M. Gene expression–based discovery of atovaquone as a STAT3 inhibitor and anticancer agent. Blood J Am Soc Hematol (2016) 128(14):1845–53. doi: 10.1182/blood-2015-07-660506 PMC5054697 27531676

[33] Verma V Jafarzadeh N Boi S Kundu S Jiang Z Fan Y. MEK inhibition reprograms CD8+ T lymphocytes into memory stem cells with potent antitumor effects. Nat Immunol (2021) 22(1):53–66. doi: 10.1038/s41590-020-00818-9 33230330 PMC10081014

[34] Hosokawa K Muranski P Feng X Townsley DM Liu B Knickelbein J. Memory stem T cells in autoimmune disease: high frequency of circulating CD8+ memory stem cells in acquired aplastic anemia. J Immunol (2016) 196(4):1568–78. doi: 10.4049/jimmunol.1501739 PMC4744506 26764034

[35] Fazeli P Talepoor AG Faghih Z Nasser Gholijani N Ataollahi MR Hassanzadeh MA. The frequency of CD4+ and CD8+ circulating T stem cell memory in type 1 diabetes. Immun Inflamm Dis (2022) 10(10). doi: 10.1002/iid3.715 PMC9500591 36169248

[36] Fujisawa R Haseda F Tsutsumi C Hiromine Y Noso S Kawabata Y. Low programmed cell death-1 (PD-1) expression in peripheral CD4+ T cells in Japanese patients with autoimmune type 1 diabetes. Clin Exp Immunol (2015) 180(3):452–7. doi: 10.1111/cei.12603 PMC4449773 25682896

[37] Zhong T Li X Zhou Z. 1175-p: circulating CD4+/CD8+ PD-1+ T cells are increased during the partial remission phase in patients with type 1 diabetes. Diabetes Care (2019) 68(Supplement_1):. doi: 10.2337/db19-1175-P

[38] Lee YJ Park JA Kwon H Choi YS Jung KC Park SH. Role of stem cell–like memory T cells in systemic lupus erythematosus. Arthritis Rheumatol (2018) 70(9):1459–69. doi: 10.1002/art.40524 29660266

[39] Cianciotti BC Ruggiero E Campochiaro C Oliveira G Magnani ZI Baldini M. CD4+ memory stem T cells recognizing citrullinated epitopes are expanded in patients with rheumatoid arthritis and sensitive to tumor necrosis factor blockade. Arthritis Rheumatol (2020) 72(4):565–75. doi: 10.1002/art.41157 31682074

[40] Takeshita M Suzuki K Kondo Y Morita R Okuzono Y Koga K. Multi-dimensional analysis identified rheumatoid arthritis-driving pathway in human T cell. Ann Rheum Dis (2019) 78(10):1346–56. doi: 10.1136/annrheumdis-2018-214885 PMC6788883 31167762

[41] Cao J Zhang C Han X Cheng H Chen W Qi K. Emerging role of stem cell memory-like T cell in immune thrombocytopenia. Scand J Immunol (2019) 89(3):. doi: 10.1111/sji.12739 30506564

[42] Schoettler ML Nathan DG. The pathophysiology of acquired aplastic anemia: current concepts revisited. Hematol Oncol Clin North Am (2018) 32(4):581–94. doi: 10.1016/j.hoc.2018.03.001 PMC6538304 30047412

[43] Zeng Y Katsanis EJC Immunology E. The complex pathophysiology of acquired aplastic anaemia. Clin Exp Immunol (2015) 180(3):361–70. doi: 10.1111/cei.12605 PMC4449765 25683099

[44] Lee RW Nicholson LB Sen HN Chan CC Wei L Nussenblatt RB. Autoimmune and autoinflammatory mechanisms in uveitis. In: Seminars in immunopathology. (Germany: Springer) (2014) 36(5):581–94. doi: 10.1007/s00281-014-0433-9 PMC4186974 24858699

[45] Pan L Lu M-P Wang J-H Yang SR. Immunological pathogenesis and treatment of systemic lupus erythematosus. World J Pediatr (2020) 16:19–30. doi: 10.1007/s12519-019-00229-3 30796732 PMC7040062

[46] Swinkels M Rijkers M Voorberg J Vidarsson G Leebeek FWG Jansen AJG. Emerging concepts in immune thrombocytopenia. Front Immunol (2018) 9:880. doi: 10.3389/fimmu.2018.00880 29760702 PMC5937051

[47] Firestein GS McInnes IB. Immunopathogenesis of rheumatoid arthritis. Immunity (2017) 46(2):183–96. doi: 10.1016/j.immuni.2017.02.006 PMC5385708 28228278

[48] Pastore F Martocchia A Stefanelli M. Hepatitis c virus infection and thyroid autoimmune disorders: a model of interactions between the host and the environment. World J Hepatol (2016) 8(2):83. doi: 10.4254/wjh.v8.i2.83 26807204 PMC4716530

[49] Lu X Song B Weng W. CD8+ stem cell-like memory T cell subset is associated with disease progression in chronic hepatitis c virus infection. Viral Immunol (2022) 36(1):25–32. doi: 10.21203/rs.3.rs-1533048/v1 36346310

[50] Asadipour M Fazeli P Zohouri M Bemani P Mohebbiniya M Khansalar S. IL-18 in blood serum of hepatitis c patients might be of predictive value for individual outcomes. Infect Disord Drug Targets (2021) 21(3):389–93. doi: 10.2174/1871526520666200707113401 32634083

[51] Silva-Junior AL Garcia NP Cardoso EC Dias S Tarragô AM Fraiji NA. Immunological hallmarks of inflammatory status in vaso-occlusive crisis of sickle cell anemia patients. Front Immunol (2021) 12:559925. doi: 10.3389/fimmu.2021.559925 33776989 PMC7990896

[52] Torres LS Okumura JV Silva DGH Mimura KKO Belini-Júnior E Oliveira RG. Inflammation in sickle cell disease: differential and down-expressed plasma levels of annexin A1 protein. PLoS One (2016) 11(11). doi: 10.1371/journal.pone.0165833 PMC5089686 27802331

